# Altered Sphingolipids, Glycerophospholipids, and Lysophospholipids Reflect Disease Status in Idiopathic Steroid-Sensitive Nephrotic Syndrome in Children: A Non-Targeted Metabolomic Study

**DOI:** 10.3390/cells14241950

**Published:** 2025-12-09

**Authors:** Takahiro Kanai, Hideo Ogiso, Jun Aoyagi, Masanori Kurosaki, Tomomi Maru, Marika Ishii, Kazuya Tanimoto, Mitsuaki Yoshino, Yuri Yamashita, Toshihiro Tajima, Ryozo Nagai, Kenichi Aizawa

**Affiliations:** 1 Department of Pediatrics, Jichi Medical University, 3311-1, Yakushiji, Shimotsuke 329-0498, Tochigi, Japan; 2 Department of Translational Research, Clinical Research Center, Jichi Medical University Hospital, 3311-1, Yakushiji, Shimotsuke 329-0498, Tochigi, Japan; 3 Jichi Medical University, 3311-1, Yakushiji, Shimotsuke 329-0498, Tochigi, Japan; 4 Clinical Pharmacology Center, Jichi Medical University Hospital, 3311-1, Yakushiji, Shimotsuke 329-0498, Tochigi, Japan

**Keywords:** sphingolipids, sphingomyelins, glycerophospholipids, lysophospholipids, pediatric ISSNS

## Abstract

Idiopathic steroid-sensitive nephrotic syndrome (ISSNS) is the most common glomerular disease in children, yet its molecular mechanisms and lipid-mediated pathophysiology remain poorly understood. In this study, we performed comprehensive non-targeted metabolomic analysis of serum samples obtained from children with ISSNS during both the nephrotic and remission phases to identify metabolic alterations associated with disease status. Using liquid chromatography–quadrupole time-of-flight mass spectrometry (LC-QTOF-MS), we profiled low-molecular-weight metabolites and identified significant alterations in several lipid classes, including sphingolipids, glycerophospholipids, and lysophospholipids. Several sphingomyelin and phosphatidylcholine species showed strong correlations with total cholesterol levels, reflecting lipid alterations consistent with the hyperlipidemic state that characterizes ISSNS. In contrast, oxidized phosphatidylcholines may more specifically reflect oxidative membrane injury and glomerular permeability changes associated with disease status. These findings highlight membrane lipid remodeling as a key feature of active disease and suggest potential lipid-based biomarkers for disease monitoring and therapeutic evaluation in pediatric ISSNS. This study provides a metabolomic framework for understanding lipid-driven mechanisms of ISSNS pathophysiology.

## 1. Introduction

Idiopathic steroid-sensitive nephrotic syndrome (ISSNS) is the most common glomerular disease in children [1]. Its incidence is 1.4–6.1 per 100,000 children [2], with a male-to-female ratio of 1.8:1 [3]. ISSNS is characterized by heavy proteinuria due to dysfunction of the glomerular filtration barrier, to hypoalbuminemia (<3.0 g/dL) caused by urinary protein loss, and to hypercholesterolemia, in the absence of underlying diseases. Even though steroid monotherapy can induce complete remission within 4 weeks [4], the mechanism by which steroids achieve this effect remains unclear [1]. Furthermore, the pathophysiology of ISSNS itself is still poorly understood. Consequently, no curative therapy has been established, and approximately 80% of patients experience relapse [4]. Therefore, understanding the pathophysiology of pediatric ISSNS is essential.

Previous studies have suggested that sera from patients with ISSNS contain pathogenic factors [5]. For example, administration of supernatant from peripheral blood mononuclear cell cultures derived from patients with minimal change nephrotic syndrome (MCNS), which typically presents as steroid-sensitive nephrotic syndrome, can induce proteinuria [6]. Furthermore, plasma apheresis treatment, such as low-density lipoprotein (LDL) apheresis and/or double-filtration plasmapheresis, can induce remission in MCNS without steroids or other medications [7].

To identify these factors, comprehensive serum analysis using non-targeted metabolomics is required. This methodology enables detection of changes in metabolite profiles between the nephrotic and remission phases of pediatric ISSNS. Liquid chromatography-quadrupole time-of-flight mass spectrometry (LC-QTOF-MS) is a powerful tool for comprehensive serum metabolomics. Metabolite profiling of sera, which has been instrumental in revealing the pathophysiology of pediatric ISSNS, can reveal distinct metabolic signatures associated with disease status.

Two studies have applied non-targeted metabolomics to investigate serum metabolite profiles associated with disease status in pediatric nephrotic syndrome. They identified metabolites and pathways related to the tricarboxylic acid cycle, amino acid metabolism, bile acid biosynthesis, linoleate metabolism, and glyoxylate and dicarboxylate metabolism [8], as well as metabolites such as D-mannitol, dulcitol, D-sorbitol, and xanthosine monophosphate [9]. However, these studies did not provide definitive insights into the pathophysiology of pediatric ISSNS. Their limitations included: (i) a heterogenous study populations (idiopathic nephrotic syndrome may include idiopathic steroid-resistant nephrotic syndrome, the pathophysiology of which differs from that of ISSNS) [8]; (ii) non-standardized timing of sample collection with or without steroid treatment; (iii) comparisons limited to sera from children with ISSNS during the nephrotic phase and those from healthy controls, without comparisons between nephrotic and remission phases [9]; and (iv) exclusive use of a normal-phase hydrophilic interaction chromatography (HILIC) [9], which is not suited to strongly non-polar compounds.

To overcome these limitations, we analyzed serum metabolite profiles using paired samples collected from pediatric ISSNS patients during both the nephrotic and remission phases, without steroid treatment. Both C_18_ and HILIC columns were employed in positive and negative ion modes to ensure comprehensive coverage.

## 2. Patients and Methods

### 2.1. Patients

Inclusion criteria were as follows: (i) pediatric ISSNS, defined as remission achieved within 4 weeks of treatment with prednisolone at 2 mg/kg/day (maximum 60 mg/day) in the absence of underlying diseases [4]; (ii) selectivity index < 0.1, measured by the clearance ratio of IgG/transferrin; (iii) no infection for 1 week prior to the study to avoid confounding effects of infection; and (iv) no immunosuppressant for 6 months prior to this study, to exclude the confounding influence of immunosuppressants. The nephrotic phase was defined as urinary protein > 300 mg/dL in a morning sample on three consecutive days [10]. The remission phase was defined as a urinary protein/creatinine ratio (UP/UCr) < 0.2 g/g·Cr on the three consecutive days [10]. Nephrotic phase samples were collected before initiation of steroid treatment or albumin administration. Remission phase samples were collected when patients were not receiving steroids or immunosuppressants. Definitions of steroid-sensitive nephrotic syndrome, nephrotic phase, and remission phase were based on IPNA clinical practice recommendations [4]. Secondary SSNS and steroid-resistant nephrotic syndrome were excluded.

A total of 22 paired samples from 14 patients (10 males, 4 females; median age 4 years, range 1–14 years) were included with samples collected during both the nephrotic and remission phases. Of these, 15 samples were collected from 7 patients during different clinical courses. Patient profiles were similar to those reported in previous studies of pediatric ISSNS, including a male/female ratio of 2.5:1 [3], and an allergic complication rate of 43% (6/14 patients; food allergy 1, asthma 2, hay fever 3) [11]. No patients had comorbidities other than the allergies described above, and none were taking anti-allergic medications at the time of blood collection. The median UP/UCr was 7.8 g/g·Cr (range, 1.7–17.6 g/g·Cr) in the nephrotic phase. The median serum albumin level was 2.7 g/dL (range, 0.7–4.0 g/dL) in the nephrotic phase, and was 4.2 g/dL (range, 2.9–4.1 g/dL) in the remission phase. The median total cholesterol level was 234 mg/dL (range, 151–520 mg/dL) in the nephrotic phase, and was 162.5 mg/dL (range, 136–294 mg/dL) in the remission phase. These differences were statistically significant (both *p* < 0.01). Serum samples were obtained from blood samples collected from patient antecubital veins and were not treated before freezing. No sample derivation was performed prior to analysis.

### 2.2. Methods

#### 2.2.1. Sample Preparation for LC-MS/MS Metabolomics

Serum samples were stored at −80 °C and thawed immediately prior to use. Fifty microliters of serum were mixed with 250 μL of methanol in a 1.5 mL polypropylene vial. After vigorous shaking, samples were centrifuged (12,000× *g* for 10 min at 4 °C), after which 50 μL of the upper layer were mixed with 200 μL of methanol/ultra-pure water (1:1, *v*/*v*) in a new 1.5 mL polypropylene vial. Samples were again centrifuged after shaking (12,000× *g* for 5 min at 4 °C), and then 200 μL of the upper layer were collected in a 0.3 mL polypropylene vial. Polypropylene vials previously washed with methanol were used for all preparations.

#### 2.2.2. Non-Targeted Metabolomic Analysis Using LC-QTOF-MS

Liquid chromatography quadrupole time-of-flight mass spectrometry (LC-QTOF-MS) analysis was performed using an LCMS9030 system (Shimadzu, Kyoto, Japan) based on a previously reported method [12] with minor modification. Serum metabolites were separated on a YMC-Accura Triart C_18_ column (100 mm × 2.1 mm, 1.9 μm; YMC Corporation, Kyoto, Japan). Mobile phases A and B consisted of 0.1% (*v*/*v*) formic acid in ultra-pure water and 0.1% (*v*/*v*) formic acid in acetonitrile/methanol (1:1, *v*/*v*), respectively. The solvent gradient was as follows: 0% B for 1 min followed by a linear gradient to 5% B from 1 to 3 min, to 90% B from 3 to 7 min, and to 100% B from 7 to 11 min, held at 100% B from 11 to 15 min. Subsequently, the mobile phase was returned to initial conditions over 0.5 min and maintained for 4.5 min until the end of the run. The oven temperature was 45 °C, and the flow rate was 0.32 mL/min. The sample volume injected was 2 μL.

For analysis of hydrophilic metabolites, LC separation was additionally performed using a normal-phase (HILIC) column (ZIC-p HILIC; 100 mm × 2.1 mm, 5 μm; SeQuant, Darmstadt, Germany). Mobile phases A and B consisted of 10 mM ammonium bicarbonate in ultra-pure water, pH 9.2, and acetonitrile/2-propanol/methanol (255:30:15, *v*/*v*/*v*), respectively. The initial buffer composition was 95% B, decreasing linearly to 60% B from 0 min to 8 min and to 5% B from 8 min to 10 min. It was then maintained at 5% B from 10 min to 12 min. Subsequently, the mobile phase was returned to the initial conditions over 0.5 min and maintained for 7.5 min until the end of the run. The oven temperature was 40 °C, and the flow rate was 0.27 mL/min. The sample volume injected was 2 μL.

MS data were acquired in both positive (pos) and negative (neg) ion modes over the usual range of 70 to 900 *m*/*z* using electrospray ionization. MS parameters were used with default settings as follows: interface voltage of 4.5 kV (pos) and −3.5 kV (neg), interface temperature of 300 °C, nebulization gas flow of 3 L/min, heating gas flow of 10 L/min, drying gas flow of 3 L/min, heat block temperature of 400 °C, and DL temperature of 250 °C. Before sample measurements, *m*/*z* values were calibrated using a calibrant (ESI Tuning Mix for Ion Trap, Sigma-Aldrich, St. Louis, MO, USA) so that mass accuracy throughout data collection was within 10 ppm. One representative among samples tested was used as a quality control (QC) sample. The QC sample was injected five times in total at the beginning, middle, and end of each batch to check stability of detection amounts during measurements (Supplementary Table S1), confirming that peak detection was stable during sample measurement. The relative abundance of each component was evaluated by the peak intensity of the parent ion (MS1) in full scan mode. Additional measurements were also made in data-dependent auto MS/MS mode to obtain fragmentation ions (MS2) necessary for structure estimation. Fragmentation was performed using argon as a collision gas at a collision energy of 30 eV with a spread of 15 eV. MS2 spectra were generated simultaneously for the top five MS1 ions with an *m*/*z* range between 70 and 900, surpassing an intensity threshold of 2000. Data were collected using LabSolutions software version 5.118 for LCMS9030 (Shimadzu).

### 2.3. Data Processing and Statistical Analysis

After converting acquired data into xml files, peak detection and alignment were performed using MS-DIAL (version 5.1) to assign peak IDs [13]. Compound names were then annotated using both MS-DIAL and MS-FINDER (version 3.56) [14]. As necessary, MS1 ions were searched against the HMDB metabolite database (version 5.0, accessed on 28 May 2025), MassBank of North America (accessed on 28 May 2025), MetFrag (accessed on 28 May 2025), and the LIPID MAPS lipid database (accessed on 28 May 2025). If MS2 spectra matched one blood metabolite, this compound was annotated as Rank A. If a significant MS2 spectrum could not be obtained, but the MS1 value matched the only metabolite registered in HMDB, it was also annotated as Rank A. If MS1 and/or MS2 spectra matched, but could not be resolved to a single compound due to multiple candidate metabolite hits, a compound that we judged most likely to be a serum metabolite was annotated as Rank B (Supplementary Figure S1).

**Figure 1:**
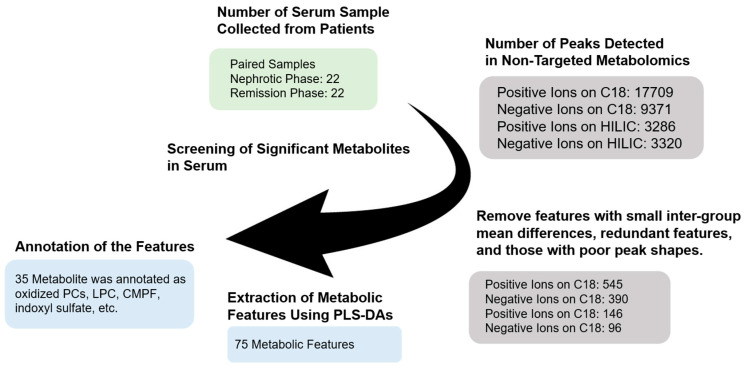
Screening Procedure for Serum Metabolites in Non-Targeted Metabolomics. Metabolomic features in serum samples were selected using various PLS-DA methods (o-PLS-DA, s-PLS-DA) to discover possible biomarkers for pediatric ISSNS.

Selection and refinement of metabolites were performed as shown in Figure 1. Over 30,000 peaks were detected by LC-QTOF-MS in serum metabolic analyses. Prior to multivariate analysis, peaks exhibiting high variability or minimal differences between the nephrotic and remission groups were manually excluded. Subsequently, important metabolic features distinguishing the two groups were selected using partial least squares discriminant analysis (PLS-DA) in MetaboAnalyst (version 6.0, accessed on 28 May 2025). Non-targeted metabolomics and extraction of characteristic components described above were performed by referring to previous reports [8,9,15,16,17]. A flow diagram summarizing the number of samples screened and analyzed is shown in Figure 1.

## 3. Results

### 3.1. Multivariate Analysis Reveals Metabolic Differences Between Nephrotic and Remission Phases

**Figure 2:**
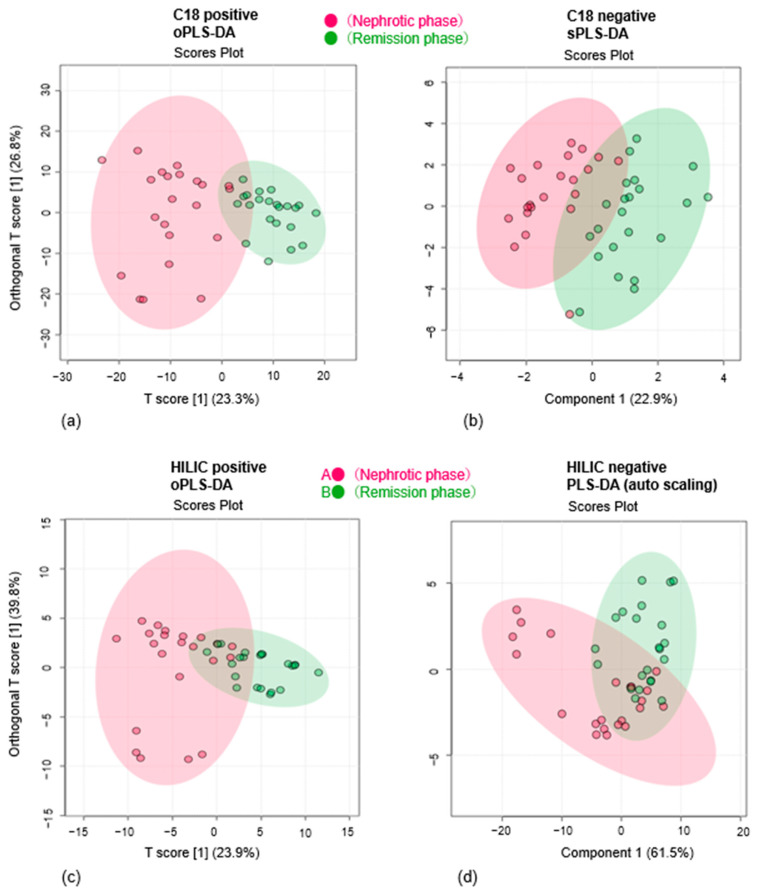
Multivariate analyses distinguishing the nephrotic and remission phases of pediatric ISSNS. (**a**) Orthogonal partial least squares discriminant analysis (oPLS-DA) using a C_18_ column in positive ion mode; (**b**) Sparse partial least squares discriminant analysis (sPLS-DA) using a C_18_ column in negative ion mode; (**c**) Orthogonal partial least squares discriminant analysis (oPLS-DA) using a HILIC column in positive ion mode; (**d**) Partial least squares discriminant analysis (PLS-DA) using a HILIC column in negative ion mode. Score plots for all analyses are shown for comparison between nephrotic and remission phases. Data points for the nephrotic phase are shown in red, and those for the remission phase are shown in green.

Score plots generated from oPLS-DA, sPLS-DA, and PLS-DA demonstrated a trend toward separation between the nephrotic and remission phases (Figure 2) when analyzed using both C_18_ and HILIC columns under positive and negative ion modes. Four representative score plots are shown in Figure 2. Multivariate analyses enabled clear discrimination between the two groups across all four measurements, revealing differences in serum metabolites. From these analyses, 75 metabolic features were initially selected as contributors to the group separation.

### 3.2. Identification of Metabolites That Differ Between Disease Phases

While primary selection was performed using PLS-DA, secondary selection for each of the 75 metabolic features involved univariate significance testing (q < 0.05) and fold-change analysis (|FC| > 1.5 or <0.5) between the two groups. Subsequent compound annotation identified 23 and 12 metabolites as Rank A and Rank B, respectively (Table 1 and Table 2). MS/MS spectrum searches for five non-lipid compounds among the 23 Rank A annotations are presented in Supplementary Figure S1. This analysis demonstrated that serum metabolites that were elevated in the nephrotic phase and reduced in remission included oxidized phosphatidylcholines (PCs), lysophosphatidylcholines (LPCs), and sphingomyelins (SMs) (Table 1), whereas those that were decreased in the nephrotic phase and elevated in remission included indoxyl sulfate and 3-carboxy-4-methyl-5-propyl-2-furanpropionic acid (CMPF), [(4-{3-[2-(2,4-dihydroxyphenyl)-2-oxoethyl]-4,6-dihydroxy-2-methoxyphenyl}[17]-2-methylbut-2-en-1-yl)oxy]sulfonic acid (DPMPS), (2-{9-hydroxy-2-oxo-2H,8H,9H-furo[2,3-h]chromen-8-yl}-2-[(3-methylbut-2-enoyl)oxy]propoxy)sulfonic acid (HOCPS), and indoxyl sulfate (Table 2). These metabolites are summarized in Table 3.

### 3.3. Correlation Analysis with Clinical Parameters

**Figure 3:**
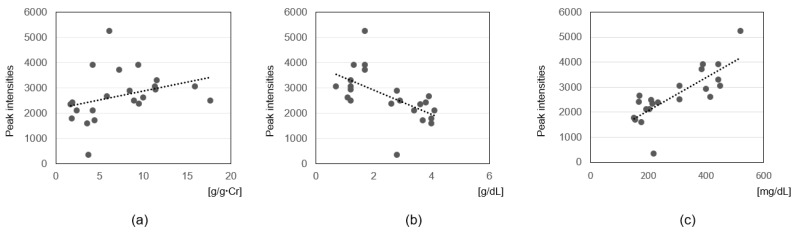
Correlations between serum sphingomyelin (d34:2) levels and clinical parameters in pediatric ISSNS. (**a**) Urinary protein-to-creatinine (UP/UCr) ratios; (**b**) serum albumin levels; (**c**) serum total cholesterol levels. Scatter plots show the relationships between sphingomyelin (d34:2) peak intensities and each clinical parameter. Spearman’s correlation coefficients (ρ) are indicated, and regression lines are shown.

**Figure 4:**
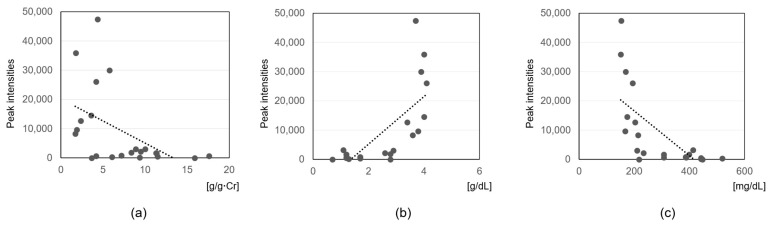
Correlations between serum 3-carboxy-4-methyl-5-propyl-2-furanpropionic acid (CMPF) levels and clinical parameters in pediatric ISSNS. (**a**) Urinary protein-to-creatinine (UP/UCr) ratios; (**b**) serum albumin levels; (**c**) serum total cholesterol levels. Scatter plots show the relationships between CMPF peak intensities and each clinical parameter. Spearman’s correlation coefficients (ρ) are indicated, and regression lines are shown.

To assess clinical relevance, Spearman’s rank correlation analysis was conducted between metabolite intensities and key clinical parameters: UP/UCr, serum albumin, and total cholesterol levels (Table 4, Figure 3 and Figure 4). For UP/UCr ratios, two metabolites including SM (d34:2) showed strong positive correlations (ρ ≥ 0.6), whereas CMPF exhibited a strong negative correlation (ρ ≤ −0.6). Correlations with serum albumin revealed strong negative associations for four metabolites including SM (d34:2) and SM (d32:1) (ρ ≤ −0.6), and strong positive associations for indoxyl sulfate, CMPF, DPMPS, and HOCPS (ρ ≥ 0.6). For serum total cholesterol, strong positive correlations were observed with six metabolites including multiple SM species and LPE (18:0) (ρ ≥ 0.6), while CMPF, DPMPS, HOCPS and indoxyl sulfate showed strong negative correlations (ρ ≤ −0.6).

## 4. Discussion

This study demonstrated that sphingolipids (sphingomyelins), glycerophospholipids (phosphatidylcholine), lysophospholipids (lysophosphatidylcholine and lysophosphatidylethanolamine), and uremic toxins (CMPF and indoxyl sulfate) reflect the disease status of ISSNS in children. Serum levels of these lipids were elevated, whereas levels of uremic toxins were decreased during the nephrotic phase compared with the remission phase. Therefore, these sphingolipids, glycerophospholipids, lysophospholipids and uremic toxins may serve as biomarkers for pediatric ISSNS. To the best of our knowledge, this is the first report to demonstrate a simultaneous association between these lipid classes, uremic toxins, and disease status of pediatric ISSNS.

There is a close metabolic relationship between sphingomyelin, phosphatidylcholine, and lysophosphatidylcholine. Sphingomyelin is generated by transferring phosphocholine from phosphatidylcholine to ceramide. Phosphatidylcholine is synthesized from lysophosphatidylcholine by lysophosphatidylcholine acyltransferase, whereas lysophosphatidylcholine is generated from phosphatidylcholine by phospholipase A_2_. Additionally, these sphingolipids, glycerophospholipids, and lysophospholipids are components of the outer leaflet of cell membranes [18], and are important in podocyte function, cholesterol homeostasis, immune regulation, and platelet activation through lipid-mediated signaling pathways [19]. Hyperlipidemia, immune dysregulation, and platelet activation are major complications of pediatric ISSNS, suggesting that lipid alterations contribute to disease pathophysiology.

### 4.1. Sphingomyelin and Proteinuria Mediated Podocyte Dysfunction

In this study, SM (d34:2) intensities in serum were positively correlated with UP/UCr ratios. Sphingolipids contribute to formation of mechanically stable and chemically resistant outer leaflets of cell membrane lipid bilayers and are an important component of lipid rafts, particularly in podocytes. These rafts are essential for podocyte signaling, membrane trafficking, and cytoskeletal organization [20]. Podocytes are terminally differentiated glomerular epithelial cells, which form the glomerular filtration barrier. Consequently, podocyte dysfunction leads to abnormal glomerular filtration, resulting in proteinuria and nephrotic syndrome. Indeed, sphingomyelin accumulation and dysfunction in podocytes reportedly causes proteinuria in Niemann–Pick disease [21,22]. Furthermore, decreased expression of sphingomyelin phosphodiesterase acid-like 3b (SMPDL-3b) has been reported in patients with pediatric idiopathic nephrotic syndrome during the nephrotic phase, compared with those in remission and with controls [23]. SMPDL-3b is expressed at the plasma membrane, particularly in podocytes, where it hydrolyzes sphingomyelin into ceramide and phosphocholine. Thus, decreased SMPDL-3b expression results in sphingomyelin accumulation and dysfunction. Because decreased SMPDL-3b expression alone does not directly impair podocyte function, sphingomyelin accumulation likely renders podocytes more susceptible to dysfunction [24], thereby contributing to proteinuria. Moreover, rituximab, a monoclonal antibody that protects against proteinuria in pediatric ISSNS, reportedly prevents decreased SMPLD-3b expression in podocytes [25]. Taken together, elevated serum sphingomyelin levels may reflect podocyte dysfunction and injury. Therefore, serum sphingomyelin levels may correlate with the degree of proteinuria and serum albumin levels.

### 4.2. Dysregulation of Cholesterol Homeostasis Induced by Sphingomyelin, Phosphatidylcholine, and Lysophosphatidylcholine

In this study, serum levels of sphingomyelin, phosphatidylcholine, and lysophosphatidylcholine were elevated during the nephrotic phase compared with the remission phase. These lipids are also components of both LDL and high-density lipoprotein (HDL) particles. Although their relative proportions vary depending on the study population and analytical methodology, LDL is generally enriched in sphingomyelin and phosphatidylcholine, whereas HDL contains predominantly phosphatidylcholine [26,27,28]. Serum LDL levels are elevated in the nephrotic phase of pediatric ISSNS compared with remission, whereas HDL levels remain unchanged [29,30]. This observation suggests that the altered serum levels of sphingomyelin, phosphatidylcholine, and lysophosphatidylcholine between the two phases are primarily attributable to LDL. In nephrotic syndrome, mechanistically, increased LDL synthesis [31] and reduced LDL catabolism due to impaired lipoprotein lipase activity [32] have been proposed as underlying mechanisms. Thus, podocyte dysfunction in pediatric ISSNS may cause proteinuria and increased serum sphingolipid concentrations, which in turn may contribute to elevated serum LDL levels.

### 4.3. Immunological Effects of Sphingomyelin, Phosphatidylcholine, and Lysophosphatidylcholine

Sphingomyelin, phosphatidylcholine and lysophosphatidylcholine affect immune regulation through T cells, B cells, and macrophages, and activation of these cells is well established as a major pathogenic mechanism of pediatric ISSNS [33,34,35].

Sphingolipid metabolism generates lipid second messengers that regulate key stages of immune cell development, differentiation, activation, proliferation, and function [36]. For example, sphingomyelin metabolism in T cells affects T cell function and development of T cell-mediated immunity [37] and both sphingomyelin and phosphatidylcholine promote growth of B lymphocytes through raftophilic species [38].

Phosphatidylcholine further promotes gamma-delta T cell activation [39] and modulates T cell responses [40]. In macrophages, phosphatidylcholine induces robust changes in gene expression, leading to their activation [41]. Moreover, lysophosphatidylethanolamine (LPE) also promotes T cell differentiation [42]. Also, sphingomyelin activates macrophages by stabilizing raft microdomains that cluster Fcγ receptors [43].

Lysophosphatidylcholine additionally activates resting T cells, leading to IL-2 production and IL-2 receptor expression [44]. Consistently, serum IL-2 levels are elevated during the nephrotic phase in pediatric ISSNS [45]. Lysophosphatidylcholine also activates B cells and enhances antibody production by modulating membrane dynamics and amplifying B cell receptor-mediated signaling. This process leads to upregulation of genes required for B cell differentiation and immunoglobulin secretion [46].

### 4.4. Phosphatidylcholine and Mechanisms of Platelet Activation

Phosphatidylcholine is a precursor of platelet activating factor (PAF) synthesis [47]. Therefore, elevated phosphatidylcholine levels may lead to increase PAF production, contributing to platelet activation and thrombus formation, which are also common complications of pediatric ISSNS [48]. Furthermore, PAF also increases vascular permeability and induces vasodilatation [49], both of which are relevant to pediatric ISSNS pathophysiology [50]. Indeed, plasma PAF concentrations are significantly higher in the nephrotic phase compared with the remission phase and control patients [51]. Sphingomyelin-rich domains in platelets provide platforms for fibrin–actomyosin interactions, thereby contributing to clot retraction [52], which is one of the complications of pediatric ISSNS. Lysophosphatidylcholine is a major component of platelet-derived microvesicles. Via its interaction with G2AR on platelets, it contributes to platelet activation and ultimately to vascular inflammation [53].

### 4.5. Association of Sphingolipids and CMPF with Clinical Parameters

This study further revealed that sphingolipid, SM (d34:2), showed strong correlation with UP/UCr ratios, serum albumin, and total cholesterol levels, all of which are closely related to pediatric ISSNS pathophysiology. As stated previously, sphingolipids are essential for maintaining structural and functional integrity of podocytes [54], injuries to which cause proteinuria. Therefore, sphingolipids may be involved not only in disease status, but also in the underlying pathophysiology of pediatric ISSNS. Podocyte dysfunction may be one of the reasons why serum sphingolipid levels were associated with these parameters; however, this cannot be confirmed based on the present findings. Nonetheless, the findings of this study collectively suggest that specific sphingolipids and related metabolites are closely associated with clinical markers of disease status, highlighting their potential role as biomarkers or contributors to the pathophysiology of pediatric ISSNS.

In addition, this study also revealed that uremic toxin CMPF (Supplementary Figure S2) shows strong negative correlations with UP/UCr ratios, serum albumin, and total cholesterol levels. CMPF is a compound that accumulates as the glomerular filtration rate (GFR) decreases and is known as one of the protein-bound uremic toxins (PBUTs) that bind almost completely (approximately 100%) to serum albumin [55,56]. Indoxyl sulfate (Supplementary Figure S3) is another uremic toxin that accumulates with reduced GFR and is a representative protein-bound uremic toxin that binds strongly (90–98%) to serum albumin [55,56]. In children with ISSNS, GFR remains within the normal range. Therefore, the negative correlation of serum CMPF and indoxyl sulfate levels with serum albumin concentration, and their positive correlation with the UP/UCr, are thought to reflect an increase in unbound CMPF and indoxyl sulfate in the nephrotic phase, when hypoalbuminemia results from urinary albumin loss. Correlations between uremic toxins and total cholesterol levels may be a secondary effect due to nephrotic condition. Therefore, these uremic toxins (CMPF and indoxyl sulfate) are likely secondary to the disease process of pediatric ISSNS rather than primary causative factors.

CPMeF (Supplementary Figure S4), a minor derivative belonging to the same family as CMPF, has been detected in blood and urine; however, its reference levels have not been established, and its physiological or pathological significance remains largely unknown (Human Metabolome Database, Version 5.0). In addition, chemical compounds of HOCPS (Supplementary Figure S5) and DPMPS (Supplementary Figure S6) are currently not identifiable in standard chemical databases such as PubChem or ChemSpider. Further investigation is required to determine their roles in pediatric ISSNS.

### 4.6. Oxidized Phosphatidylcholine and Pediatric ISSNS

Increased levels of oxidized phosphatidylcholine (OxPC) species during the nephrotic phase suggest an association with the disease status of pediatric ISSNS. OxPCs themselves can directly cause endothelial dysfunction, independent of oxidized LDL-mediated mechanisms [57]. Children with FRNS and SDNS have endothelial dysfunction during relapse, which is largely dependent upon disease status [58]. Moreover, their accumulation may represent a functional lipid alteration contributing to the pathophysiology of pediatric ISSNS [59]. This finding supports the notion that oxidative modification of membrane lipids may exacerbate pediatric ISSNS [60].

Additionally, oxidized LDL, which contains phosphatidylcholine and sphingomyelin as major lipid components, can also cause endothelial dysfunction that may lead to proteinuria [61].

Overall, multivariate and correlation analyses provide robust evidence that metabolic alterations are phase-dependent, reproducible across chromatographic and ionization conditions, and closely linked to disease-relevant clinical parameters. Integration of these findings enables identification of key lipid classes and metabolites with potential mechanistic significance in pediatric ISSNS.

## 5. Limitations

This study has several limitations. First, intensities of sphingolipids, glycerophospholipids and lysophospholipids were not compared with those in other forms of nephrotic syndrome or in healthy controls. Therefore, it cannot be concluded that the increases observed are specific to pediatric ISSNS. Indeed, Erkan et al. reported increased lysophosphatidylcholines in patients with FSGS [62]. Second, this study was not designed as an intervention trial to evaluate effects of these lipid classes in pediatric ISSNS. Thus, we cannot assert that the observed associations cause pediatric ISSNS. Third, the small sample size (n = 14 patients) is a major limitation that affects the generalizability and statistical power of the study. Finally, in this study, non-targeted metabolomic analysis was employed for exploratory purposes. However, implementation of LC-MS-based metabolomics and lipidomics in routine clinical settings faces several challenges. The most critical issue is the lack of inter-laboratory standardization, as results may vary among institutions. To ensure reproducibility and clinical applicability, establishment of standardized targeted aLC-MS methods using stable isotope-labeled internal standards will be essential in the future.

## 6. Conclusions

Sphingolipids, glycerophospholipids, and lysophospholipids appear to be involved in pediatric ISSNS disease status. These lipid classes are linked to hyperlipidemia, immune dysregulation, and platelet activation, and may thereby contribute to the pathophysiology of pediatric ISSNS. Further investigations are needed to clarify underlying mechanisms and pathogenic processes of this disease.

## Figures and Tables

**Table 1:** Annotated metabolites and putatively annotated compounds with increased intensity in the nephrotic phase of pediatric ISSNS (fold change > 1.5, q ≤ 0.05 vs. remission phase).

Annotated Compounds	FC	*p* Values	q Values	Adduct Type	*m*/*z*	LC Mode	RT (min)
**PC(16:0/18:2)+O**	5.599	0.026802	0.044286	[M+HCOO]^−^	818.563	C18	11.281
**PC(16:0/22:6)+2O**	5.049	0.025874	0.041429	[M+HCOO]^−^	882.557	C18	11.096
**PC(18:0/18:2)+O**	4.207	0.017478	0.04	[M+HCOO]^−^	846.593	C18	12.076
**LPC(17:0)**	3.038	0.003903	0.028571	[M+HCOO]^−^	554.351	C18	9.3
**LPC(18:0)**	2.329	0.002104	0.024286	[M+HCOO]^−^	568.367	C18	9.479
**PC(16:0/20:3)**	2.319	0.007069	0.034286	[M+HCOO]^−^	828.583	C18	13.633
**LPE(18:0)**	1.848	0.002407	0.027143	[M−H]^−^	480.313	C18	9.679
**PC(16:0/22:4)**	1.685	0.010441	0.037143	[M+HCOO]^−^	854.597	C18	13.608
**LPC(20:2)**	1.661	0.000149	0.011429	[M+HCOO]^−^	592.367	C18	9.22
**LPC(16:0)**	1.639	0.000252	0.014286	[M+HCOO]^−^	540.335	C18	8.819
SM(d34:2)	1.608	0.000136	0.008571	[M+HCOO]^−^	745.556	C18	12.654
SM(d33:1)	1.577	0.000323	0.015714	[M+HCOO]^−^	733.556	C18	12.697
PC(18:0/20:2)	1.576	0.000141	0.01	[M+HCOO]^−^	858.631	C18	15.066
PC(35:1)	1.54	0.000085	0.007143	[M+HCOO]^−^	818.599	C18	14.435
SM(d34:1)	1.534	0.000483	0.018571	[M+HCOO−C2H4O2]^−^	687.55	C18	12.976
PC(18:1/20:3)	1.522	0.006156	0.031429	[M+HCOO]^−^	854.598	C18	13.454
SM(d32:1)	1.519	0.001116	0.022857	[M+HCOO]^−^	719.541	C18	12.257
PC(38:3)	1.5	0.006332	0.032857	[M+HCOO]^−^	856.605	C18	13.455
ΔFA(18:1)+3O	7.738	0.046204	0.05	[M−H]^−^	329.236	C18	7.358
ΔcPA(16:0)	5.693	0.005325	0.03	[M−H]^−^	391.228	C18	8.987
ΔPyroglutamylglycine	3.304	0.012598	0.038571	[M+H]^+^	187.073	HILIC	2.902
ΔGlutamylglutamate	2.405	0.000032	0.002857	[M+H]^+^	277.109	HILIC	0.961
ΔPyroglutaminylglutamine or Methylcytidine	2.24	0.035083	0.048571	[M+H]^+^	258.111	HILIC	3.043
ΔPyroglutamylglycine	2.204	0.028601	0.047143	[M+H]^+^	187.073	HILIC	2.766
Δc-Glutamylglutamic acid	1.858	0.008448	0.035714	[M+H]^+^	277.104	HILIC	6.346
ΔAsparaginylvaline	1.751	0.02629	0.042857	[M+H]^+^	232.131	HILIC	3.044
ΔPC(34:2)	1.693	0.028382	0.045714	[M+H]^+^	758.616	C18	13.249
ΔSM(d18:1/16:0)+O	1.636	0.000041	0.004286	[M+HCOO]^−^	763.568	C18	11.783
ΔPC(36:2)	1.545	0.002177	0.025714	[M+H]^+^	786.607	C18	13.409

**Legend**: The top 10 metabolites (by absolute fold change among those with q ≤ 0.05 in Rank A) are highlighted in bold. Δ denotes metabolites annotated as Rank B. q-values were calculated using the Benjamini–Hochberg false discovery rate (FDR) procedure to adjust for multiple comparisons among the 35 tested metabolites. **Abbreviations**: FC, fold change (mean nephrotic/remission ratio); *p* value, *p* value from Welch’s *t*-test; LC mode, separation mode in liquid chromatography; RT, retention time; PC, phosphatidylcholine; LPC, lysophosphatidylcholine; LPE, lysophosphatidylethanolamine; SM, sphingomyelin; FA, fatty acid; cPA, cyclic phosphatidic acid; PC(16:0/22:6)+2O, oxidized form of PC(16:0/22:6) with two additional oxygen atoms.

**Table 2:** Annotated metabolites and putatively identified compounds showing decreased intensity in the nephrotic phase of ISSNS (fold change < 0.5, q ≤ 0.05 vs. nephrotic phase).

Annotated Compounds	FC	*p* Values	q Values	Adduct Type	*m*/*z*	LC Mode	RT (min)
**CMPeF**	0.249	0.00001	0.0014286	[M−H]^−^	267.1245	C18	7.879
**CMPF**	0.264	0.000066	0.0057143	[M−H]^−^	239.094	C18	7.376
**DPMPS**	0.267	0.000549	0.02	[M−H]^−^	453.088	HILIC	2.581
**HOCPS**	0.34	0.000557	0.0214286	[M−H]^−^	439.072	HILIC	2.822
**Indoxyl sulfate**	0.492	0.000337	0.0171429	[M−H]^−^	212.004	C18	5.606
ΔPDMS	0.461	0.000151	0.0128571	[M+H]^+^	536.17	C18	12.809

**Legend**: Boldface indicates metabolites annotated as Rank A with q ≤ 0.05. Δ denotes metabolites annotated as Rank B. **Abbreviations**: FC, fold change (mean nephrotic/remission ratio); *p* value, *p*-value from Welch’s *t*-test; LC mode, separation mode in liquid chromatography; RT, retention time; CMPeF, 3-carboxy-4-methyl-5-pentyl-2-furanpropionic acid; CMPF, 3-carboxy-4-methyl-5-propyl-2-furanpropionic acid; DPMPS, [(4-{3-[2-(2,4-dihydroxyphenyl)-2-oxoethyl]-2,4,6-trihydroxyphenyl}-2-methylbut-2-en-1-yl)oxy]sulfonic acid; HOCPS, (2-{9-hydroxy-2-oxo-2H,8H,9H-furo[2,3-h]chromen-8-yl}-2-[(3-methylbut-2-enoyl)oxy]propoxy)sulfonic acid; PMDS, polydimethylsiloxane.

**Table 3:** Classification of lipid classes and urinary toxins, with abbreviations and full names.

	Classification	Full Names	Abbreviation
Lipid classes	Sphingolipids	Sphingomyelin	SM
	Glycerophospholipids	Phosphatidylcholine	PC
	Lysophospholipids	Lysophosphatidylcholine	LPC
		Lysophosphatidylethanoamine	LPE
Uremic toxins		3-carboxy-4-methyl-5-propyl-2-furanpropionic acid	CPMF
		Indoxyl sulfate	

**Table 4:** Spearman’s correlations between metabolite intensities and urinary protein-to-creatinine (UP/UCr) ratio, serum albumin, and total cholesterol levels in pediatric ISSNS.

Annotated Compounds	FC	Correlation Efficient		
		UP/UCr	Serum Alb	Serum T.chol
**SM(d34:2)**	1.608	0.611	−0.648	0.676
**LPE(18:0)**	1.848			0.606
**SM(d33:1)**	1.577			0.684
**SM(d34:1)**	1.534			0.632
**SM(d32:1)**	1.519		−0.634	0.698
**CMPeF**	0.249	−0.725	0.831	−0.752
**CMPF**	0.264	−0.606	0.747	−0.836
**DPMPS**	0.267		0.768	−0.725
**HOCPS**	0.340		0.679	−0.657
**Indoxyl sulfate**	0.492		0.719	
ΔSM(d18:1/16:0)+O	1.636	0.646	−0.624	0.699
ΔGlutamylglutamate	2.405		−0.606	

**Legend**: Boldface indicates metabolites annotated as Rank A in pediatric ISSNS. Δ denotes metabolites annotated as Rank B. Spearman’s correlation coefficients were calculated between metabolite intensities and UP/UCr, serum albumin, and total cholesterol levels. Positive correlations indicate higher metabolite levels associated with increased clinical parameter values, whereas negative correlations indicate inverse relationships. **Abbreviations**: FC, fold change (mean nephrotic/remission ratio); UP/UCr, urinary protein/creatinine ratio; Alb, albumin; T.chol, total cholesterol; SM, sphingomyelin; LPE, lysophosphatidylethanolamine; CMPeF, 3-carboxy-4-methyl-5-pentyl-2-furanpropionic acid; CMPF, 3-carboxy-4-methyl-5-propyl-2-furanpropionic acid; DPMPS, [(4-{3-[2-(2,4-dihydroxyphenyl)-2-oxoethyl]-4,6-dihydroxy-2-methoxyphenyl}-2-methylbut-2-en-1-yl)oxy]sulfonic acid; HOCPS, (2-{9-hydroxy-2-oxo-2H,8H,9H-furo[2,3-h]chromen-8-yl}-2-[(3-methylbut-2-enoyl)oxy]propoxy)sulfonic acid; PMDS, polydimethylsiloxane; PMDS, polydimethylsiloxane; SM(d18:1/16:0)+O, oxidized form of SM(d18:1/16:0) with one additional oxygen atom.

## Data Availability

The original contributions presented in this study are included in the article/Supplementary Materials. Further inquiries can be directed to the corresponding author.

## Supplementary Materials

The following supporting information can be downloaded at: https://www.mdpi.com/article/10.3390/cells14241950/s1, Figure S1: How to annotate as Rank A and B title; Figure S2: Spectrum of CMPF and the structural formula; Figure S3: Spectrum of Indoxyl sulfate and the structural formula; Figure S4: Spectrum of CPMeF and the structural formula; Figure S5: Spectrum of HOCPS and the structural formula; Figure S6: Spectrum of DPMPS and the structural formula. Table S1: Reproducibility of detection peaks for representative metabolites in QC samples.
