# Supplementary material for: Altered Sphingolipids, Glycerophospholipids, and Lysophospholipids Reflect Disease Status in Idiopathic Steroid-Sensitive Nephrotic Syndrome in Children: A Non-Targeted Metabolomic Study
Source: Cells. 2025 Dec 9;14(24):1950. doi: 10.3390/cells14241950 (PMC12732284; doi:10.3390/cells14241950)
Supplement: Supplementary file 1 [file cells-14-01950-s001.zip › cells-3984171-supplementary.pdf]

Supplementary Table S1. Reproducibility of detection peaks for representative metabolites in QC samples

LC mode	Ion	Compounds	*m*/*z*	RT (min)	Peak Intensities RSD (%) (n=5)
C18	pos	Inosine	269.093	5.059	6.145
		PC(32:1)	732.559	13.131	2.110
	neg	Inosine	267.075	5.052	5.251
		PC(32:1)	776.558	13.134	3.266
HILIC	pos	Citrulline	176.105	5.605	3.040
		Arginine	175.121	8.966	2.935
	neg	Citrulline	174.088	5.639	8.107
		Arginine	173.104	9.008	3.639

How to Annotate as Rank A and B

Both cases where MS2 spectra are obtained and not

↓

Database Search

① MS-DIAL
Compounds hit in MS-DIAL database search

② MS-FINDER
Compounds hit (with a score >5.0 if an MS2 spectrum is obtained) in multiple databases search including HMDB, ChEBI, COCONUT, MINE, KNApSAcK, etc.

③ Private Database
Compounds hit in a private list of MS1 and retention times for bile acids, fatty acids, phospholipids, and sphingolipids etc.

④ Other tools available on the web
MetFrag, MassBank of North America

Comprehensively annotated including estimation from known compounds

↓ yes

Well-known metabolites in human fluids are hit

↓ yes

Consistent with hydrophobicity estimated from its LC retention time

↓ yes

Limited to one metabolite

↓ yes

Annotated as Rank A

If no single metabolite could be selected, the most likely candidate was annotated as Rank B.
Even if a single compound was selected, it was annotated as Rank B if it is not known as a human metabolite.

Supplementary Figure S1

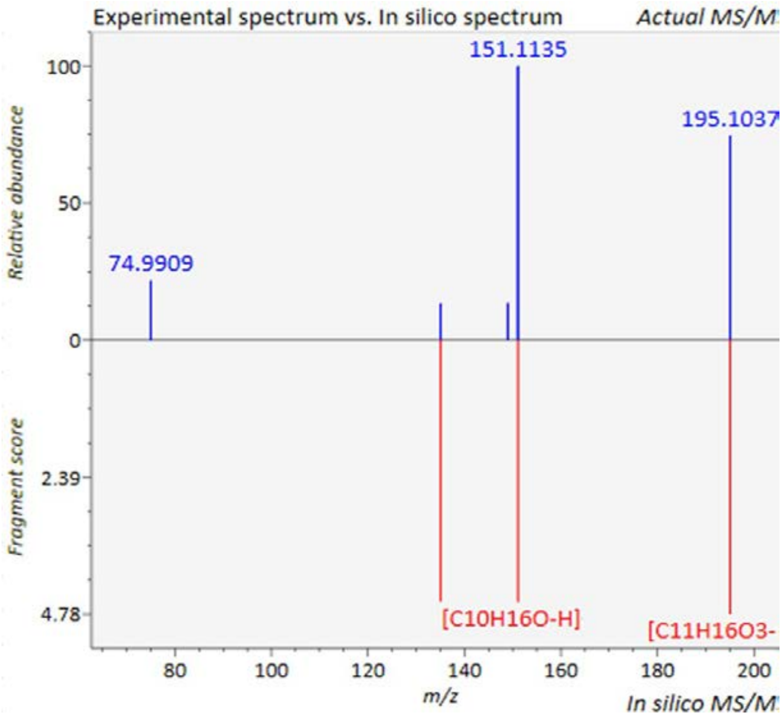

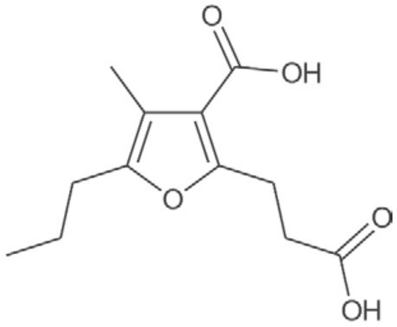

C12H16O5

3-Carboxy-4-methyl-5-propyl-2-furanpropionic acid (CMPF)

Supplementary Figure S2

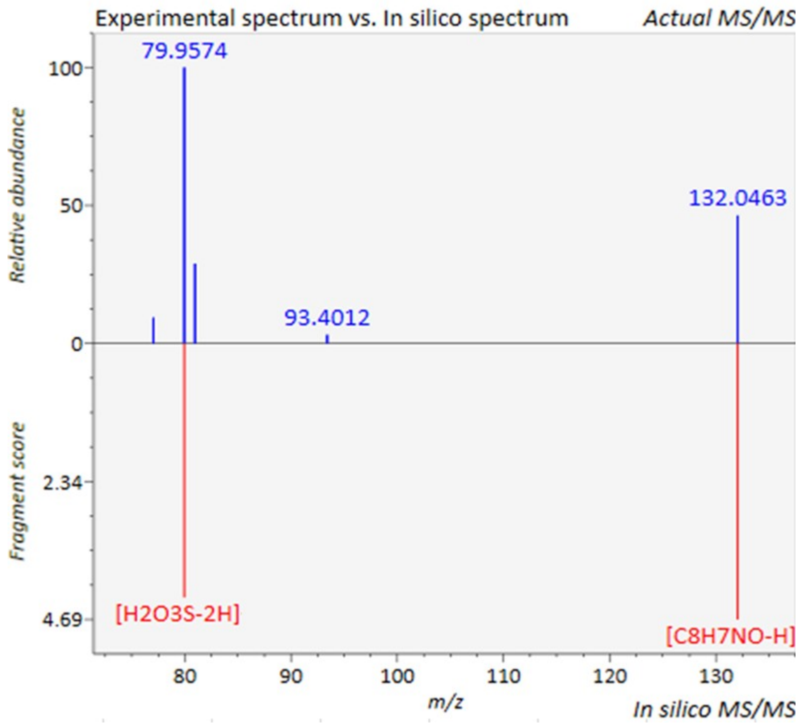

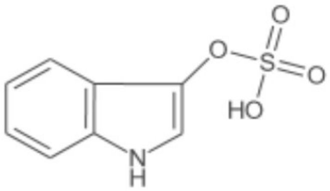

C8H7NO4S

Indoxyl sulfate

Supplementary Figure S3

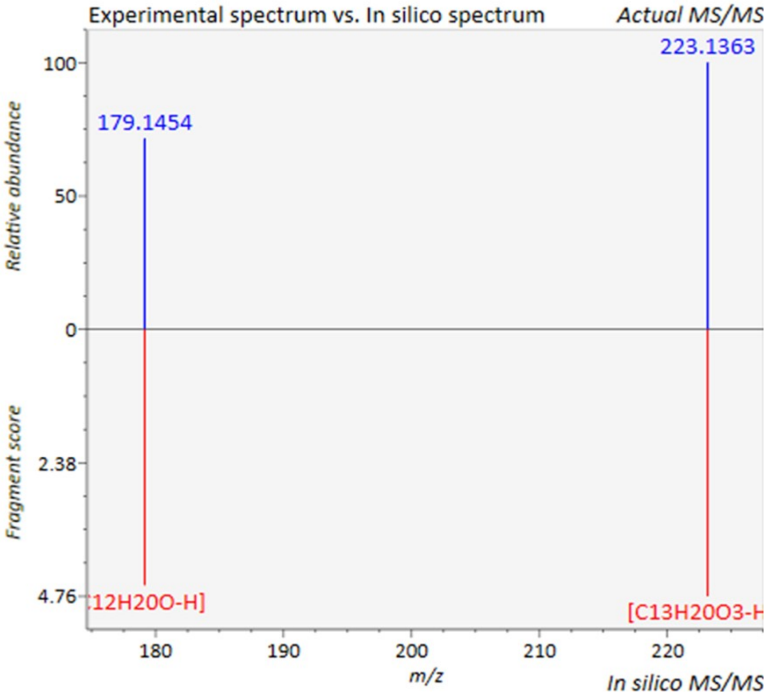

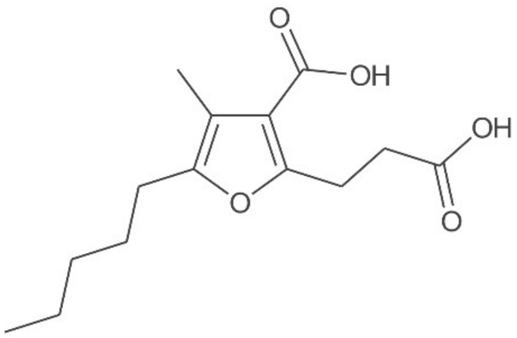

C14H20O5

3-carboxy-4-methyl-5-pentyl-2-furanpropanoic acid (CMPeF)

Supplementary Figure S4

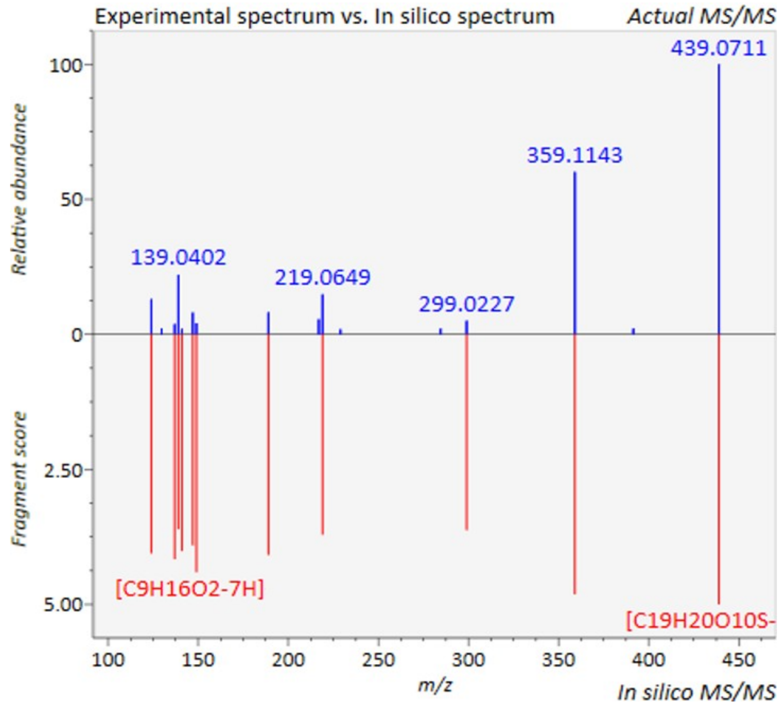

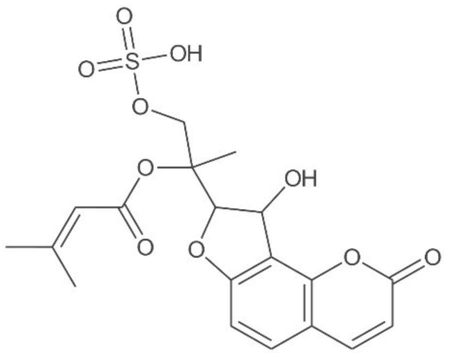

C19H20O10S

(2-{9-hydroxy-2-oxo-2H,8H,9H-furo[2,3-h]chromen-8-yl}-2-[(3-methylbut-2-enoyl)oxy]propoxy)sulfonic acid (HOCPS)

CHEBI:193287

Supplementary Figure S5

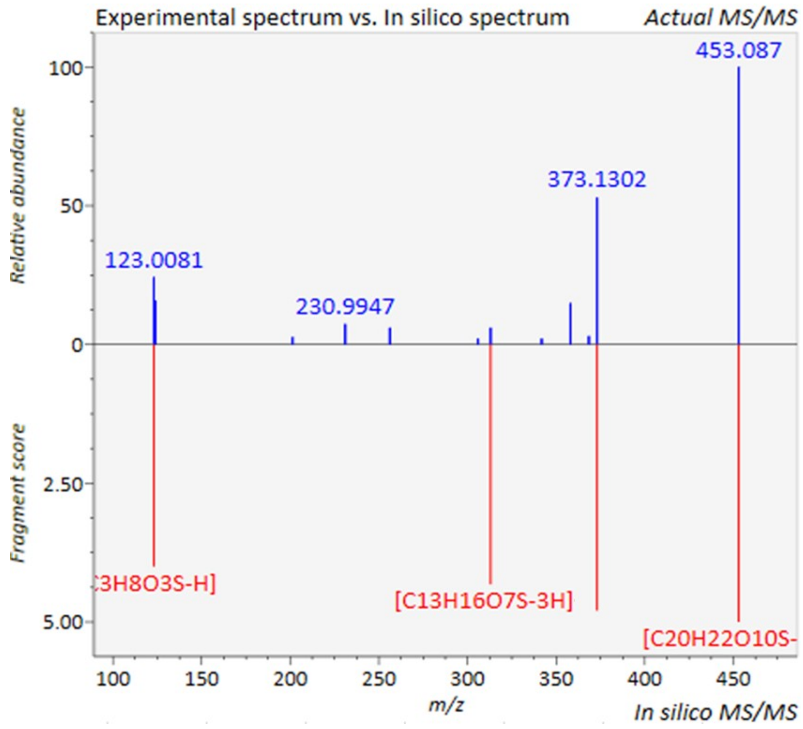

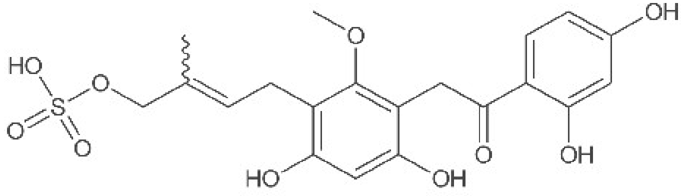

C20H22O10S

[(4-{3-[2-(2,4-dihydroxyphenyl)-2-oxoethyl]-4,6-dihydroxy-2-methoxyphenyl}-2-methylbut-2-en-1-yl)oxy]sulfonic acid (DPMPS)

CHEBI:180705

Supplementary Figure S6
